# European Psychiatry 2020: Moving forward

**DOI:** 10.1192/j.eurpsy.2019.3

**Published:** 2020-01-14

**Authors:** Andrea Fiorillo, Sophia Frangou

**Affiliations:** 1 Department of Psychiatry, University of Campania “L. Vanvitelli”, Naples, Italy; 2 Centre for Brain Health, University of British Columbia, Canada; 3 Department of Psychiatry, Icahn School of Medicine at Mount Sinai

*European Psychiatry* is the official journal of the European Psychiatric Association. Launched in 1986 by Patrice Boyer, Julien-Daniel Guelfi, and Yves Lecrubier, *European Psychiatry* has achieved a dynamic presence in the field by publishing cutting-edge clinical, biological and psychosocial research, by disseminating key policy and guidance documents, and by stimulating and fostering debate among all stakeholders in mental health and neuroscience.

*European Psychiatry* is a peer-reviewed journal that is committed to publish the latest advances in the full range of issues related to mental health, including new developments in diagnosis and treatment, and advances in the biological underpinnings of mental, behavioral, and cognitive function in clinical and general population samples. Each year, *European Psychiatry* receives an average of 600 manuscripts, mostly from Europe, North and Central America, Australia, and Asia, which attract more than 1 million annual article views and downloads. We offer authors timely feedback with a mean time to editorial desk decision of less than a week and 4 weeks with peer review. Accepted articles are published online immediately upon receipt of corrected proofs, with an average time from submission to publication of 18 weeks.

We are pleased to announce that *European Psychiatry* is now undertaking a number of changes in line with our commitment to accessibility and transparency in publishing. From January 2020, *European Psychiatry* will become a fully gold open access journal, while maintaining its high standards in peer review and adhering to the current best practices in publishing. The move to a full open access model is motivated by our commitment to the principles outlined in Plan S—that the pursuit of science is supported by free access to research for all, including the public and those in disadvantaged regions.

At the same time, the journal will move to an online only publication model, emphasizing accessibility for readers and speed to publication for authors, and will allow authors to retain copyright through the adoption of a Creative Commons Attribution (CC BY) license. We are also adopting a new data transparency policy for all submissions made to the journal.

In order to further support our goals, *European Psychiatry* is introducing a new 21-member editorial board of international experts in mental health and neuroscience with equal gender representation. Our board will have a key role in increasing the profile and influence of the journal and shaping our development strategy going forward.

The journal will also change its publisher. We are delighted to become part of the publishing family of Cambridge University Press, a not-for-profit publishing house, which shares our commitment to psychiatry and open access and our vision for the future of the journal.

As we move forward, we believe that *European Psychiatry* will not only continue to make fundamental contributions to the field of psychiatry, but also help strengthen communication and collaboration among clinicians, researchers, policy makers, and patient advocates.

